# The efficacy and safety of paravertebral block for postoperative analgesia in renal surgery: A systematic review and meta-analysis of randomized controlled trials

**DOI:** 10.3389/fsurg.2022.865362

**Published:** 2022-07-18

**Authors:** You Zhao, Yanan Kan, Xin Huang, Ming Wu, Weiping Luo, Jun Nie

**Affiliations:** ^1^ Department of Urology, People’s Hospital of Liyang City, Liyang, China; ^2^ Department of Orthopedic, The First Affiliated Hospital, Zhejiang Chinese Medical University, Hangzhou, China

**Keywords:** paravertebral block, postoperative analgesia, opioid, renal surgery, pain scores, efficacy, safety

## Abstract

**Background:**

Paravertebral block (PVB) has been widely used in postoperative analgesia, especially in thoracic and breast surgery. However, the efficacy and safety of PVB for analgesia after renal surgery remains uncertain. Therefore, this study aimed to determine the postoperative analgesic efficacy and safety of PVB in renal surgery.

**Methods:**

PubMed, Web of Science, Embase, and the Cochrane Library databases were systematically searched up to December 20, 2021. All randomized controlled trials (RCTs) evaluating the postoperative analgesic efficacy of PVB in renal surgery were collected. The meta-analysis was performed using RevMan 5.4 and Stata/MP 14.0 software.

**Results:**

A total of 16 RCTs involving 907 patients were included in the meta-analysis. Ten studies investigated patients under percutaneous nephrolithotomy (PCNL), and six studies were done for patients under other renal surgery (nephrectomy or pyeloplasty). Compared with control groups (no block, sham block, or other nerve blocks), meta-analysis showed that PVB reduced 24-hour postoperative opioid consumption significantly (SMD = −0.99, 95%CI: −1.60–0.38, *p *= 0.001, *I*^2^ = 92%) and reduced pain scores at various time points within 24 h at rest and 1 h, 4 h, and 24 h at movement after renal surgery, furthermore, PVB prolonged the time to first postoperative analgesic requirement (SMD = 2.16, 95%CI: 0.94–3.39, *p *= 0.005, *I*^2^ = 96%) and reduced the incidence of postoperative additional analgesia (OR = 0.14, 95%CI: 0.06∼0.33, *p *< 0.00001, *I*^2^ = 50%). Subgroup analysis revealed that the postoperative analgesia effect of PVB was more significant in PCNL, and the use of bupivacaine for PVB seemed to have a better performance. Besides, there was no difference in the incidence of postoperative nausea, vomiting, and itching between PVB and control groups.

**Conclusion:**

This study indicates that PVB may provide effective postoperative analgesia in patients under renal surgery, especially PCNL patients. Moreover, PVB is a safe analgesic method without significant analgesia-related complications.

## Introduction

Postoperative pain is one of the most common and troublesome problems faced by patients undergoing various operations (1). Evidence showed that less than half of patients who undergo surgery reported adequate relief of postoperative pain, while about 75% reported moderate, severe, or extreme pain (2). Timely and effective postoperative pain management has always been a big concerned problem for surgeons and patients, as inadequate pain relief could lead to a prolonged hospital stay, affect recovery progress, and increase medical costs, even cause serious complications such as cardiovascular accidents and thrombosis (3). Renal surgery, especially in open nephrectomy and PCNL is accompanied by severe postoperative pain (4). At present, continuous epidural techniques, intravenous, or oral non-opioid analgesics, quadriceps lumborum block, and nerve block are widely used for postoperative pain management after renal surgery, of which epidural block is the most commonly used, and the analgesic effect of these methods are acceptable (5, 6). However, some patients still develop chronic pain (7).

Paravertebral block (PVB) is a new analgesic method with an easy procedure, reliable curative effect, and few complications (8). Earlier studies indicated that PVB shows a significant analgesic effect for many surgical procedures; especially cardiothoracic surgery, breast surgery, and inguinal hernia repair (9–11). The application of PVB in renal surgery has been reported recently, and some studies have shown that PVB can effectively control the pain after renal surgery and reduce anesthetic-related complications (12). Therefore, the purpose of this study is to evaluate the effect of PVB on pain management after renal surgery and provide a reference for the selection of analgesic methods after renal surgery.

## Methods

### Search strategy

This study was performed by following the preferred reporting items for systematic reviews and meta-analysis (PRISMA) statement (13). Two reviewers independently and comprehensively searched PubMed, Web of Science, Embase, and the Cochrane Library databases from inception to December 20, 2021. The following search terms were used: PVB and PCNL or nephrectomy or pyeloplasty or renal surgery. The search strategies for all databases are presented in the Supplementary Material. We also manually searched the references of all the included studies to identify other relevant articles. There was no language limitation in the literature search.

### Inclusion and exclusion criteria

The inclusion criteria were defined by the population, intervention, comparison, outcome, and study design (PICOS) principle, see Table 1. The exclusion criteria of this study were: (1) duplicate articles; (2) studies were published as comments, reviews, case reports, letters, or conference abstracts; (3) studies with incomplete data; and (4) low-quality literature.

**Table 1:** PICOS framework.

Component	Description
Population	Patients undergoing renal surgery
Intervention	Paravertebral block
Comparison	No block, sham block, or other nerve blocks
Outcomes	Postoperative opioid consumption, pain scores, time to first postoperative analgesic requirement, postoperative additional analgesia, and complication
Study design	Randomized controlled trials

### Data extraction and quality assessment

Two investigators independently completed data extraction and quality assessment, simultaneous cross-checking was conducted, and any disputes were solved by discussion or third-party intervention. The following relevant information was extracted from included articles: first author, publication year, country, number of patients, type of surgery, American Society of Anesthesiologists (ASA) score, age, gender, duration of operation, anesthesia time, interventions, follow-up time, and study outcomes. The primary outcomes were 24-hour postoperative opioid consumption, and pain scores at rest and movement at 1 h, 4 h, 6 h, 12 h, and 24 h after surgery. The secondary outcomes were the incidence of postoperative additional analgesia, time to first postoperative analgesic requirement, and the incidence of postoperative nausea, vomiting, and itching. The quality of the included studies was evaluated using the Cochrane Collaboration Risk of Bias Tool (14).

### Statistical analysis

We used Review Manager Version 5.4 (Cochrane collaboration, the Nordic Cochrane Centre, Copenhagen, Denmark) and Stata/MP version 14.0 to perform the meta-analysis. For dichotomous outcomes, such as the incidence of postoperative additional analgesia and nausea, vomiting, and itching were presented as odds ratios (OR) at 95% confidence interval (CI). For continuous outcomes, such as 24-hour postoperative opioid consumption, pain scores, and time to first postoperative analgesic requirement were expressed as the mean difference (MD) at 95% CI. If the measurement units were different, standardized mean difference (SMD) with 95%CI was used. The chi-squared test and I^2^ statistics were used to evaluate heterogeneity across studies, and *I*^2^ > 50% and/or *p* < 0.1 represented significant heterogeneity. The random-effects model was applied to all analyzes to account for the clinical and methodological diversity among included studies. Sensitivity analysis was used to explore the stability of the results, and subgroup analysis was performed to investigate the effect of surgery type and anesthetic type on the results when high heterogeneity was detected. Funnel plots were used to evaluate publication bias where there were more than 10 studies. Begg and Eggers tests were further used to evaluate the asymmetry of funnel plots, and trim-and-fill analysis was performed to explore the impact of publication bias on the interpretation of the results. When *p *< 0.05, the difference was considered statistically significant.

## Results

### Search results and study characteristics

**Figure 1:**
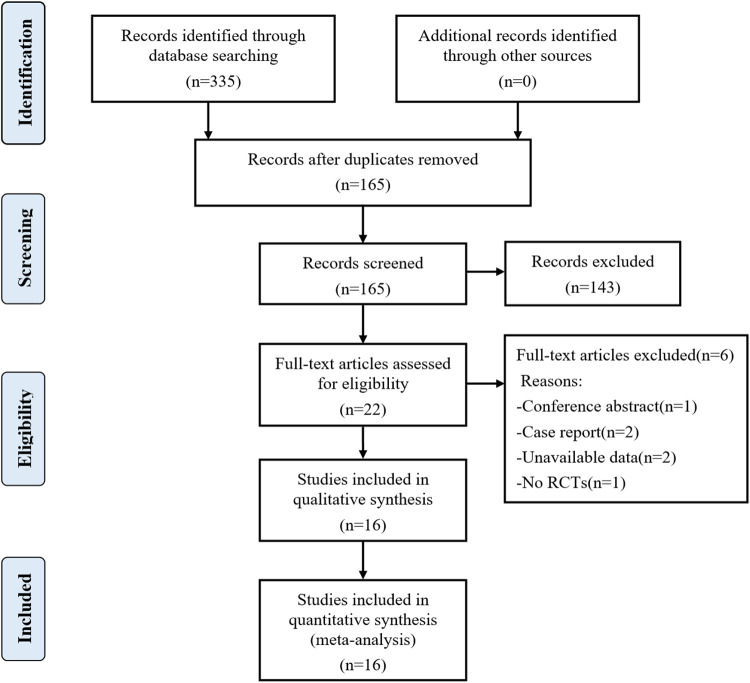
Flowchart of the study selection.

A total of 335 articles were initially searched. Finally, 16 randomized controlled trials (RCTs) involving 907 patients were included in the meta-analysis after reading full texts (15–30). The literature screening flowchart is shown in Figure 1. The baseline characteristics of the 16 RCTs are summarized in Table 2. All the 16 RCTs were published from 2013 to 2020, and the sample size was between 30 and 100. Ten RCTs (15, 16, 18, 19, 21–24, 27, 28) explored the effect of PVB in PCNL patients, five RCTs (17, 20, 25, 29, 30) were nephrectomy patients, and other one RCT (26) was pyeloplasty patients. The analgesic efficacy of PVB was compared with no block or sham block in 12 RCTs (15–22, 24, 27–29), and the other 4 RCTs (23, 25, 26, 30) compared the PVB with epidural block.

**Table 2:** Characteristics of included studies.

References	Country	Type of surgery	ASA	Groups (No. of patients)	Age (years)	Gender (M/F)	Duration of operation (min)	Methods	Postoperative analgesia	Outcomes
AK et al. (15)	Turkey	PCNL	I-II	PVB (27)	48.8 ± 9.9	15/12	52.3 ± 15.2	4 ml of 0.5% levobupivacaine inject at each of the T10, T11, and T12 paravertebral spaces	Morphine PCA and intramuscular diclofenac sodium if VAS score above five out of 10.	24-hour postoperative opioid consumption, time to first postoperative analgesic requirement, postoperative additional analgesia, postoperative nausea, vomiting, and itching
				Control (28)	50.6 ± 9.6	14/14	53.5 ± 15.9	4 ml of 0.9% NaCl solution inject at same level (sham block)		
Akinci et al. (16)	Turkey	PCNL	I-II	PVB (20)	2.77 ± 1.24		58.65 ± 18.97	A one-third dose of 0.5 ml/kg of bupivacaine 0.5% inject at each of the vertebral levels T11, T12, and L1	Tramadol 1 mg/kg iv	Pain scores, postoperative additional analgesia
				Control (20)	3.30 ± 1.40		61.15 ± 18.99	No block		
Baik et al. (17)	Korea	Open nephrectomy	I-II	PVB (17)	56 ± 10	12/5	140 ± 32.3	A single 18-ml injection of 0.75% ropivacaine at T10 or T11 paravertebral space	Fentanyl PCA	24-hour postoperative opioid consumption, pain scores, postoperative nausea
				Control (17)	55.9 ± 10.3	12/5	155 ± 56.2	No block		
Baldea et al. (18)	United States	PCNL	I-II	PVB (22)	58.2 ± 10.9	9/13	69.4 ± 34.9	A single injection of 20 ml of 0.5% bupivacaine at T10 paravertebral space	Morphine PCA or fentanyl PCA, and intravenous fentanyl and oral Norco (tylenol–hydrocodone) if needed	24-hour postoperative opioid consumption, pain scores, time to first postoperative analgesic requirement, postoperative additional analgesia, postoperative vomiting
				Control (23)	49.9 ± 11.8	12/11	72.2 ± 37.2	No block		
Borle et al. (19)	India	PCNL	I-II	PVB (24)	36.28 ± 11.95	18/6	70.4 ± 23.93	20 ml of 0.5% bupivacaine inject at the T9–10 paravertebral space	Fentanyl PCA and tramadol 50 mg iv if needed	24-hour postoperative opioid consumption, time to first postoperative analgesic requirement, postoperative additional analgesia, postoperative vomiting
				Control (24)	34 ± 13.01	16/8	76.52 ± 30.18	No block		
Copik et al. (20)	Poland	Open nephrectomy or open nephron-sparing surgery	I-III	PVB (27)	59 ± 9	17/10	91 ± 39	Plain bupivacaine 0.3 ml/kg inject at the T7 to T10 level	Oxycodone PCA and 1 g intravenous paracetamol every 6 h and 100 mg of intravenous ketoprofen every 12 h	24-hour postoperative opioid consumption, pain scores
				Control (31)	61 ± 10	20/11	85 ± 36	No block		
Hatipoglu et al. (21)	Turkey	PCNL	I-II	PVB (26)	41.8 ± 12.3	16/10	87 ± 36.3	0.5% bupivacaine for a total dose of 15 ml inject at the T11- L1 levels	Tramadol PCA and intramuscular diclofenac sodium if VAS score >4.	24-hour postoperative opioid consumption, postoperative additional analgesia, postoperative nausea and vomiting
				Control (27)	44.5 ± 14	17/10	94.1 ± 27.8	No block		
Kamble et al. (22)	India	PCNL	I-II	PVB1 (30)	44.07 ± 9.85	20/10	1.63 ± 0.58 h	0.5% bupivacaine, maximum dose 2 mg/kg, maximum volume 15 ml or 18 ml inject at T11-T12 level	Tramadol (1 mg/kg) iv at the VAS of >4.	Time to first postoperative analgesic requirement
				PVB 2 (30)	40.17 ± 11.93	17/13	1.62 ± 0.54 h	0.5% bupivacaine±1 µg/kg of clonidine		
				Control (30)	38.70 ± 11.89	18/12	1.78 ± 0.22 h	No block		
Li et al. (23)	China	PCNL	I-II	PVB (50)	48.3 ± 11.8	34/16	64.5 ± 19.4	15 ml of 0.75% ropivacaine inject into the T11 paravertebral space	Sufentanil PCA	24-hour postoperative opioid consumption, time to first postoperative analgesic requirement, postoperative nausea
				Control (50)	47.7 ± 12.2	31/19	68.5 ± 22.3	15 ml of 0.75% ropivacaine inject into the L1–L2 epidural space		
Maheshwari et al. (24)	India	PCNL	I-III	PVB (30)	38.3 ± 14.29	17/13		15 ml of 0.25% ropivacaine	Injection diclofenac sodium (50 mg) or injection paracetamol (100 mg) if VAS score > 4	Pain scores, postoperative additional analgesia
				Control (30)	37.6 ± 11.63	14/16		No block		
Moawad et al. (25)	Egypt	Open renal surgery	I-II	PVB (40)	43.57 ± 10.56	25/15	175.00 ± 35.00	1.5 mg/kg of bupivacaine 0.5% inject into the T10 paravertebral space	Intramuscular pethidine 50–100 mg if VAS score > 4	24-hour postoperative opioid consumption, postoperative nausea
				Control (40)	43.70 ± 11.22	25/15	189.00 ± 39.00	1–1.5 mg/kg of bupivacaine 0.5% inject into T10 interspace		
Narasimhan et al. (26)	India	Pyeloplasty	I-II	PVB (25)	6.0 ± 2.6	19/6	98.2 ± 22.8	0.5 ml/kg of 0.2% ropivacaine with 1:200000 adrenaline inject into the T10 paravertebral space	Fentanyl 1 μg/kg if needed	24-hour postoperative opioid consumption, pain scores, time to first postoperative analgesic requirement, postoperative additional analgesia, postoperative nausea
				Control (25)	5.1 ± 2.6	20/5	99.6 ± 20.7	Caudal block give with 1.25 ml/kg of 0.2% ropivacaine with 1:200000 adrenaline, by in-plane approach		
Yaman et al. (27)	Turkey	PCNL	I-III	PVB (22)	50.3 ± 10.5	16/6		20 ml 0.25% bupivacaine as a single administration inject into level T8-T9	Dexketoprofen 50 mg iv if VAS score > 4 or tramadol 1 mg/kg	Pain scores, postoperative additional analgesia
				Control (22)	48.7 ± 14.1	10/12		No block		
Yayik et al. (28)	Turkey	PCNL	I-II	PVB (20)	48.95 ± 13.65	11/9	126.00 ± 61.27	Single-shot paravertebral block with 20 ml 0.25% bupivacaine at the level of T8–T9	Fentanyl PCA, and 25 mg pethidine if VAS scores ≥4	24-hour postoperative opioid consumption, pain scores, postoperative additional analgesia, postoperative nausea, vomiting, and itching
				Control 1 (20)	48.65 ± 12.01	13/7	118.25 ± 59.27	Infiltration along the nephrostomy tube 20 ml 0.25% bupivacaine, in 6 and 12 o’clock positions.		
				Control 2 (20)	45.25 ± 12.09	12/8	115.75 ± 49.76	No block		
Yenidünya et al. (29)	Turkey	Donor nephrectomy	–	PVB (14)	55.5 ± 10.7	8/6	190.4 ± 46.8	0.1 ml/kg of 0.5% bupivacaine inject into T11–T12 paravertebral space	Bupivacaine PCA or morphine PCA and oral paracetamol + codeine phosphate + caffeine if the NRS score >4	24-hour postoperative opioid consumption, postoperative additional analgesia, postoperative nausea and vomiting
				Control (16)	51.0 ± 8.5	7/9	193.5 ± 50.4	No block		
Zhang et al. (30)	China	Nephrectomy	I-II	PVB (30)	44 ± 6	14/16	82 ± 5	30 ml of 0.375% ropivacaine inject into the T7 paravertebral space	Morphine PCA	Pain scores, postoperative nausea, vomiting, and itching
				Control (30)	45 ± 7	15/15	82 ± 6	15 ml of 0.375% ropivacaine inject into the T8–T9 epidural space		

### Risk of bias assessment

**Figure 2:**
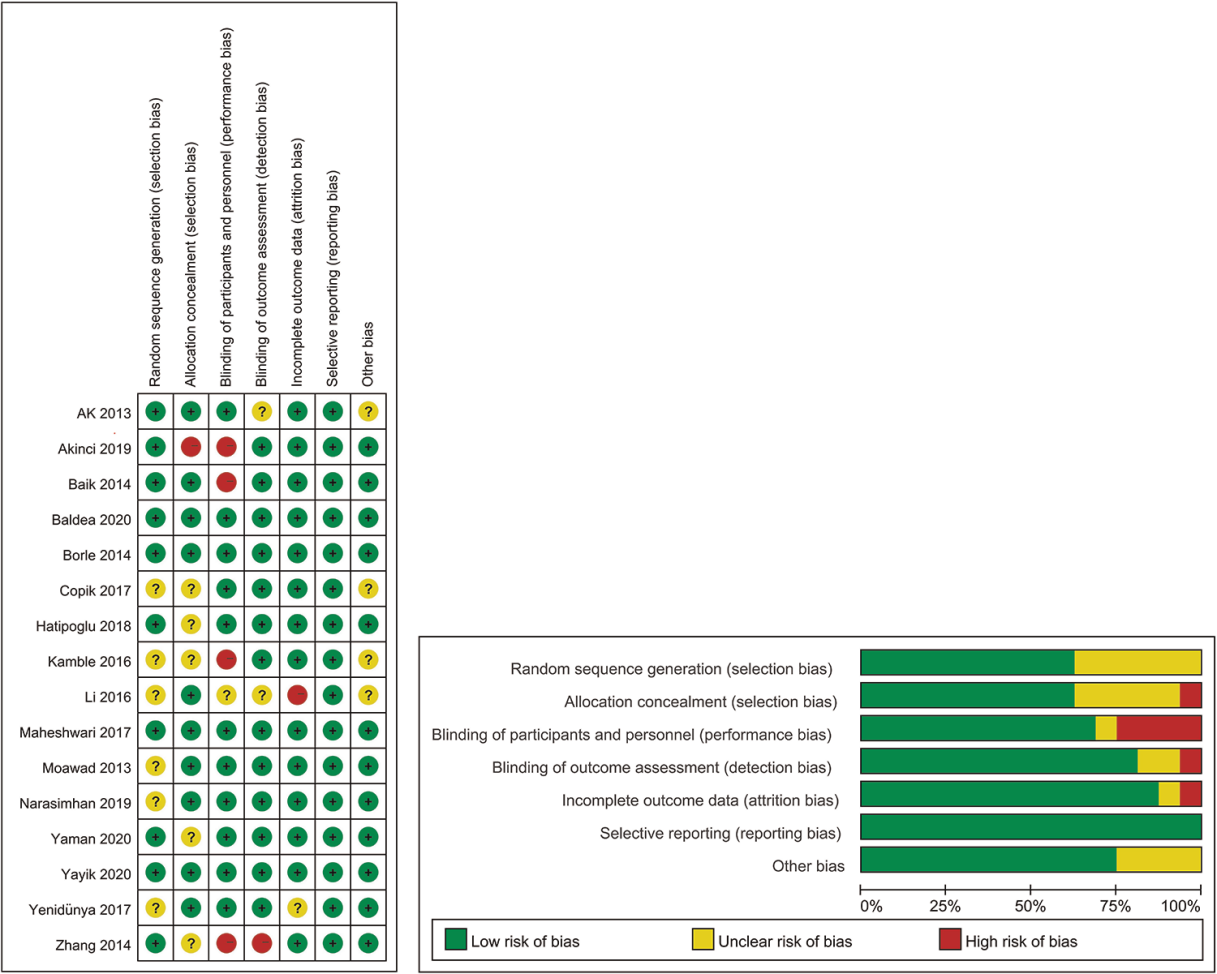
Risk of bias summary and risk of bias graph for the included studies.

The results of bias risk assessment for all RCTs are shown in Figure 2. All the included studies were rated as medium- or high-quality, and no low-quality literature was included.

### Primary outcomes

#### 24-hour postoperative opioid consumption

**Figure 3:**
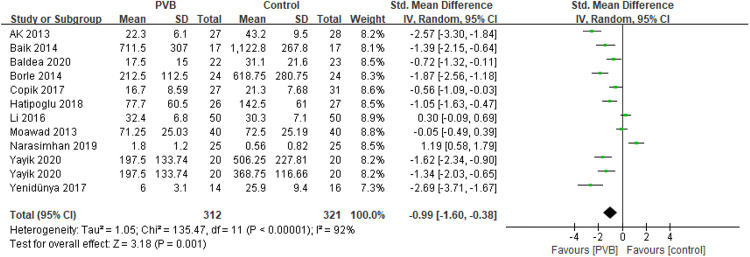
Forest plot of 24-hour postoperative opioid consumption.

Eleven RCTs (15, 17–21, 23, 25, 26, 28, 29) involving 613 patients investigated the efficacy of PVB on 24-hour postoperative opioid consumption compared with control groups. Results showed a positive effect of PVB on 24-hour postoperative opioid consumption with a large effect size compared with control groups (SMD = −0.99, 95%CI: −1.60∼−0.38, *p *= 0.001), despite substantial heterogeneity between individual study estimates (*I*^2 ^= 92%) (Figure 3). This result was robust on leave-one-out sensitivity analysis. Subgroup analysis based on surgery type and anesthetic type showed that the results were consistent with the overall findings in PCNL (SMD = −1.24, 95%CI: −2.02–0.46, *p *= 0.002, *I*^2^ = 91%) and bupivacaine (SMD = −1.33, 95%CI: −1.91–0.76, *p* < 0.00001, *I*^2^ = 87%) subgroups, while there was no significant difference in other renal surgery (SMD = −0.64, 95%CI: −1.66–0.38, *p *= 0.22, *I*^2^ = 93%) and ropivacaine (SMD = 0.06, 95%CI: −1.17–1.28, *p* = 0.93, *I*^2^ = 93%) subgroups (Table 3).

**Table 3:** Details of subgroup analysis.

Variable	Groups	No. of studies	No. of patients		Effect (95%CI)	*p-*value	Heterogeneity	
			PVB	control			*I*^2^(%)	*p* value
24-hour postoperative opioid consumption								
Surgery type	PCNL	6	189	192	SMD −1.24 (−2.02 to −0.46)	0.002	91	<0.00001
	Other renal surgery	5	123	129	SMD −0.64 (−1.66 to 0.38)	0.22	93	<0.00001
Anesthetic type	Bupivacaine	8	220	229	SMD −1.33 (−1.91 to −0.76)	<0.00001	87	<0.00001
	Ropivacaine	3	92	92	SMD 0.06 (−1.17 to 1.28)	0.93	93	<0.00001
Pain scores at rest at 1 h								
Surgery type	PCNL	3	82	82	SMD −1.19 (−1.66 to −0.72)	<0.00001	48	0.12
	Other renal surgery	3	69	73	SMD −1.22 (−2.52 to 0.07)	0.06	91	<0.00001
Anesthetic type	Bupivacaine	4	109	113	SMD −1.25 (−1.62 to −0.87)	<0.00001	39	0.16
	Ropivacaine	2	42	42	SMD −1.13 (−3.39 to 1.14)	0.33	95	<0.0001
Pain scores at rest at 6 h								
Surgery type	PCNL	3	72	72	SMD −1.17 (−1.86 to −0.48)	0.0009	73	0.03
	Other renal surgery	3	72	72	SMD −0.61 (−1.43 to 0.21)	0.15	82	0.004
Anesthetic type	Bupivacaine	2	42	42	SMD −0.82 (−1.26 to −0.37)	0.0003	0	0.93
	Ropivacaine	4	102	102	SMD −0.94 (−1.84 to −0.03)	0.04	89	<0.00001
Pain scores at rest at 12 h								
Surgery type	PCNL	3	82	82	SMD −0.31 (−0.62 to −0.00)	0.05	0	0.48
	Other renal surgery	4	99	103	SMD −0.82 (−1.29 to −0.35)	0.0007	61	0.05
Anesthetic type	Bupivacaine	4	109	113	SMD −0.48 (−0.86 to −0.09)	0.01	50	0.09
	Ropivacaine	3	72	72	SMD −0.73 (−1.35 to −0.12)	0.02	68	0.04
Time to first postoperative analgesic requirement								
Anesthetic type	Bupivacaine	4	133	135	SMD 2.87 (1.42 to 4.33)	0.0001	95	<0.00001
	Ropivacaine	2	75	75	SMD 0.45 (−1.01 to 1.91)	0.54	94	<0.00001
Incidence of postoperative additional analgesia								
Surgery type	PCNL	8	211	214	OR 0.11 (0.04 to 0.26)	<0.00001	38	0.11
	Other renal surgery	2	39	41	OR 0.37 (0.05 to 2.62)	0.32	76	0.04
Anesthetic type	Bupivacaine	8	195	200	OR 0.17 (0.06 to 0.44)	0.0003	52	0.03
	Ropivacaine	2	55	55	OR 0.08 (0.02 to 0.36)	0.0009	36	0.21

*PCA, patient-controlled analgesia; VAS, visual analog scale, NRS, numerical rating scale*.

#### Postoperative pain scores at rest and movement

**Figure 4:**
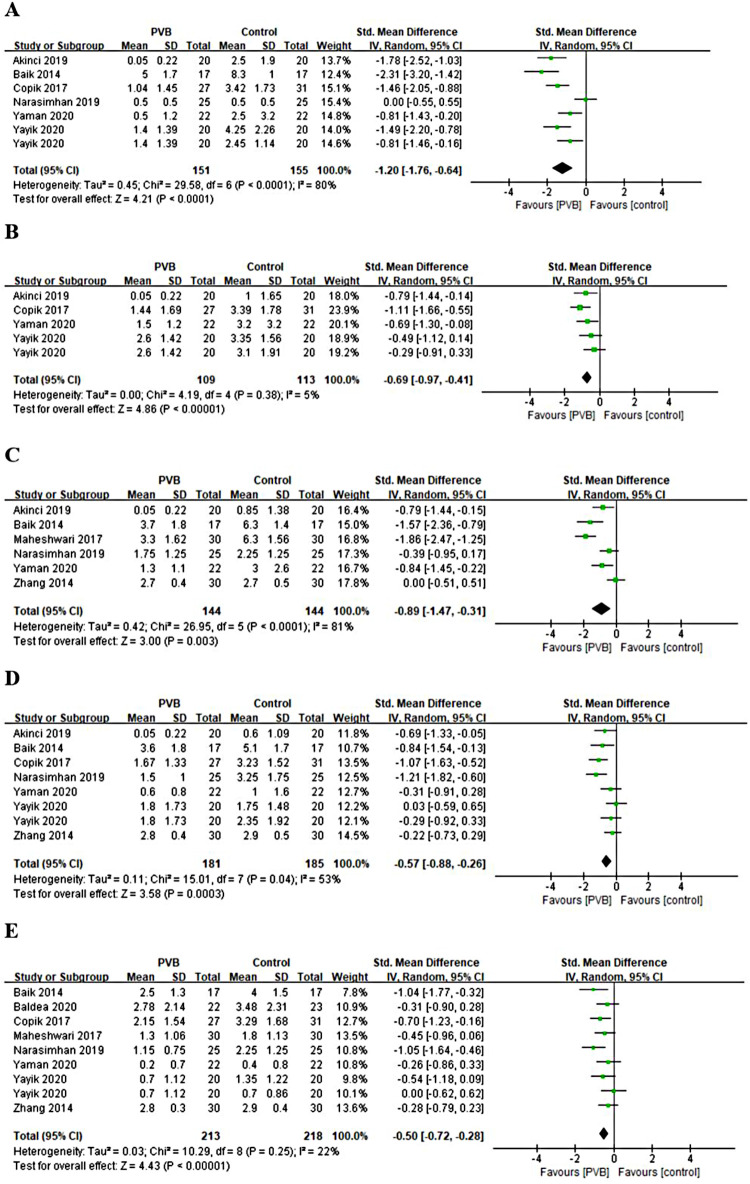
Forest plot of pain scores at rest at (**A**) 1 h, (**B**) 4 h, (**C**) 6 h, (**D**) 12 h, and (**E**) 24 h timepoints after surgery.

Nine RCTs (16–18, 20, 24, 26–28, 30) reported pain scores at different time points at rest after renal surgery using PVB. Meta-analysis showed that the PVB significant reduced pain scores at 1 h (SMD = −1.20, 95%CI: −1.76–0.64, *p *< 0.0001, *I*^2^ = 80%), 4 h (SMD = −0.69, 95%CI: −0.97–0.41, *p *< 0.00001, *I*^2^ = 5%), 6 h (SMD=−0.89,95%CI:−1.47–0.31,*p *= 0.003, *I*^2^ = 81%), 12 h (SMD = −0.57, 95%CI: −0.88–0.26, *p *= 0.0003, *I*^2^ = 53%), and 24 h (SMD = −0.50, 95%CI: −0.72–0.28, *p *< 0.00001, *I*^2^ = 22%) at rest postoperatively compared with control groups (Figures 4A–E). The results were stable on sensitivity analysis. Then subgroup analysis was carried out to explore whether pain scores at 1 h, 6 h, and 12 h at rest with high heterogeneity were affected by surgery type and anesthetic type. The results revealed that the postoperative pain scores of the PCNL and bupivacaine subgroups at 1 h, 6 h, and 12 h at rest were consistent with the overall outcomes. Similarly, the same results were observed at 12 h at rest in the other renal surgery subgroup and at 6 h, 12 h at rest in the ropivacaine subgroup, but no significant differences were found at 1 h, 6 h at rest in the other renal surgery subgroup and at 1 h at rest in ropivacaine subgroup (Table 3).

**Figure 5:**
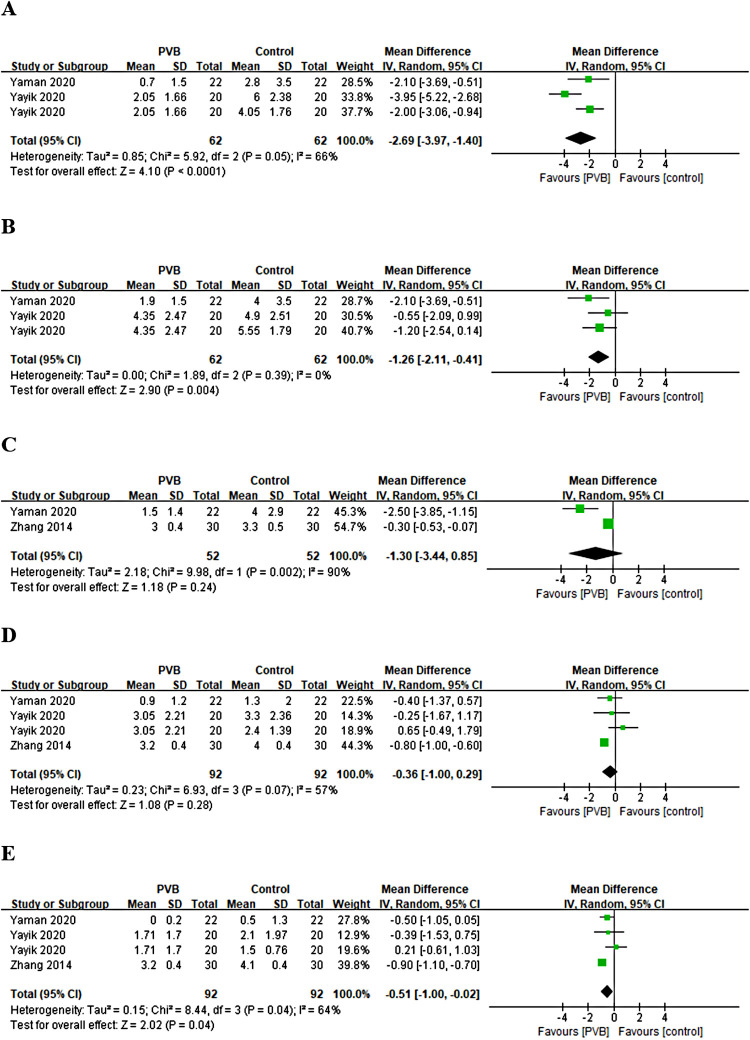
Forest plot of pain scores at movement at (**A**) 1 h, (**B**) 4 h, (**C**) 6 h, (**D**) 12 h, and (**E**) 24 h timepoints after surgery.

Only three RCTs (27, 28, 30) reported pain scores at different time points at movement after renal surgery with the use of PVB. Meta-analysis showed that the PVB significantly reduced pain scores at 1 h (MD = −2.69, 95%CI: −3.97–1.40, *p *< 0.0001, *I*^2^ = 66%), 4 h (MD = −1.26, 95%CI: −2.11–0.41, *p *= 0.004, *I*^2^ = 0%), and 24 h (MD = −0.51, 95%CI: −1.00–0.02, *p *= 0.04, *I*^2^ = 64%) at movement after surgery compared with control groups, but no significant differences were found at 6 h (MD = −1.30, 95%CI: −3.44–0.85, *p *= 0.24, *I*^2^ = 90%) and 12 h (MD = −0.36, 95%CI: −1.00–0.29, *p *= 0.28, *I*^2^ = 57%) (Figures 5A–E). The results did not change significantly on sensitivity analysis. The subgroup analysis was not performed because of the limited number of RCTs.

### Secondary outcomes

#### Time to first postoperative analgesic requirement

**Figure 6:**
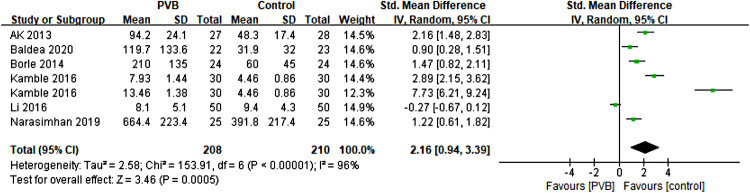
Forest plot of time to first postoperative analgesic requirement.

Six RCTs (15, 18, 19, 22, 23, 26) involving 388 patients compared the time to first postoperative analgesic requirement between PVB and control groups. Meta-analysis showed that PVB prolonged the time to first postoperative analgesic requirement significantly compared with control groups (SMD = 2.16, 95%CI: 0.94–3.39, *p *= 0.0005, *I*^2^ = 96%) (Figure 6). The result was robust on sensitivity analysis. Since all but one of the six studies was PCNL surgery, subgroup analysis based on surgery type was not performed. Subgroup analysis based on anesthetic type showed that the result of the bupivacaine subgroup was consistent with the overall findings (SMD = 2.87, 95%CI: 1.42–4.33, *p *= 0.0001, *I*^2^ = 95%), but no significant difference was found in ropivacaine subgroup (SMD = 0.45, 95%CI: −1.01–1.91, *p *= 0.54, *I*^2^ = 94%) (Table 3).

#### Incidence of postoperative additional analgesia

**Figure 7:**
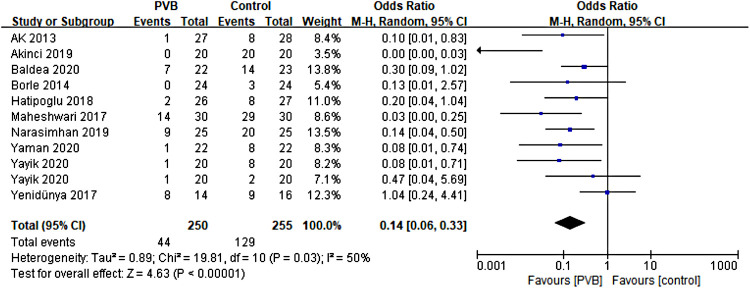
Forest plot of the incidence of postoperative additional analgesia.

Ten RCTs (15, 16, 18, 19, 21, 24, 26–29) including 485 patients reported the number of patients using additional analgesia after surgery. Meta-analysis revealed that PVB reduced the incidence of postoperative additional analgesia significantly (OR = 0.14, 95%CI: 0.06–0.33, *p *< 0.00001, *I*^2^ = 50%) (Figure 7). The result was stable on sensitivity analysis. Subgroup analysis based on surgery type revealed that the result of the PCNL subgroup was consistent with the overall outcomes (OR = 0.11, 95% CI: 0.04–0.26, *p *< 0.00001, *I*^2^ = 38%), while no significant difference was found in another renal surgery subgroup (OR = 0.37, 95% CI: 0.05∼2.62, *p *= 0.32, *I*^2^ = 76%). We also performed subgroup analysis based on anesthetic type, and significant differences were found in both bupivacaine (OR = 0.17, 95%CI: 0.06–0.44, *p* = 0.0003, *I*^2^ = 52%) and ropivacaine (OR = 0.08, 95%CI: 0.02–0.36, *p* = 0.0009, *I*^2^ = 36%) subgroups (Table 3).

#### Incidence of postoperative nausea, vomiting, and itching

**Figure 8:**
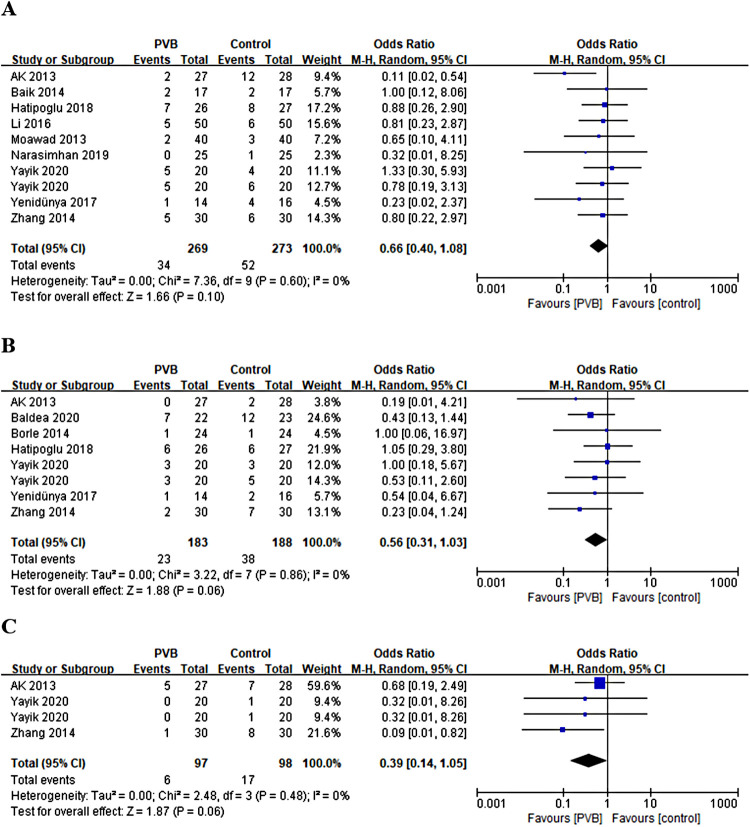
Forest plot of the incidence of (**A**) nausea, (**B**) vomiting, and (**C**) itching.

Nine RCTs (15, 17, 21, 23, 25, 26, 28–30) involving 522 patients reported the number of patients with postoperative nausea. No statistically significant difference was shown in the incidence of postoperative nausea between the PVB and the control group (OR = 0.66, 95%CI: 0.40–1.08, *p *= 0.10, *I*^2^ = 0%) (Figure 8A). Seven RCTs (15, 18, 19, 21, 28) involving 351 patients reported the number of patients with postoperative vomiting. The result revealed no significant difference in the incidence of postoperative vomiting as well (OR = 0.56, 95%CI: 0.31–1.03, *p *= 0.06, *I*^2^ = 0%) (Figure 8B). Three RCTs involving 175 patients reported the number of patients with postoperative itching. There was also no significant difference in the incidence of postoperative itching between PVB and control group (OR = 0.39, 95%CI: 0.14–1.05, *p *= 0.06, *I*^2^ = 0%) (Figure 8C). Sensitivity analysis confirmed that these results were robust.

#### Publication bias

**Figure 9:**
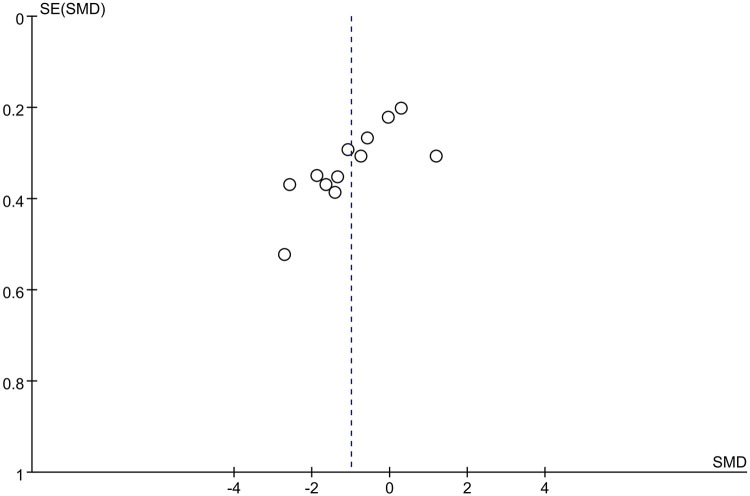
Funnel plot of evaluating publication bias.

A funnel plot was drawn based on the comparison of 24-hour postoperative opioid consumption, the distribution of the included studies was not symmetrical and there was a possibility of publication bias (Figure 9), which was also confirmed by Begg and Egger tests (*p* = 0.007, *p* = 0.004). However, the trim-and-fill analysis indicated that the results of this study were not affected by publication bias.

## Discussion

This study demonstrated that PVB may be an ideal analgesic method, which can improve the postoperative analgesia of patients undergoing renal surgery significantly. In particular, early postoperative opioid consumption and pain scores were reduced remarkably. Further, the first postoperative analgesia time was prolonged, and the postoperative additional analgesia rate was reduced. Meanwhile, the PVB did not increase the incidence of postoperative nausea, vomiting, and itching compared to other controls.

The basic principle of PVB is to inject local anesthetics into both sides of the vertebral body and near the spinal nerve roots out of the intervertebral foramen, in order to achieve the analgesic effect by blocking the paravertebral spinal nerve (31). In a comparative study as early as 1993, continuous PVB showed better postoperative pain relief than continuous epidural block after renal surgery (32). In recent years, clinical studies indicated that PVB can be used for postoperative analgesia in patients undergoing renal surgery (33). Saroa et al. (34) reported that ipsilateral PVB guided by a single ultrasound, whether levobupivacaine or ropivacaine, can provide adequate and effective analgesia after PCNL. In other renal surgeries, such as nephrectomy, PVB also has excellent postoperative analgesia reported by Tomar et al. (35). Similarly, PVB can effectively alleviate the pain of patients with various types of renal surgery at most time points in the early postoperative period, and the result of this meta-analysis was consistent with them. Among the 16 RCTs included in this study, except one Li et al. (23), pointed out that PVB can relieve postoperative pain, especially in the early rest state after surgery. Our integrated analysis showed that PVB could significantly reduce pain scores at various time points within 24 h at rest and at 1 h, 4 h, and 24 h at movement after renal surgery, while no significant difference was observed at 6 h and 12 h at movement. Then, sensitivity analysis was performed by leave-one-out method and found that these results were robust. Considering that different renal surgeries and anesthetics may have an impact on postoperative pain, we conducted a subgroup analysis based on surgery type and anesthetic type. A summary of the results showed that PVB appeared to reduced postoperative pain scores more significantly in PCNL, and PVB using bupivacaine was more effective.

A previous meta-analysis of the analgesic effect of PVB in PCNL showed that PVB could reduce postoperative analgesic consumption and the use of additional analgesics, and prolong the time required for the first analgesia, but had no substantial effect on nausea, vomiting, and itching (36). This meta-analysis, which included all newly published RCTs of various types of renal surgery using PVB, showed that PVB significantly reduced 24-hour postoperative opioid consumption. Subgroup analysis showed that the results were statistically significant differences in PCNL and bupivacaine subgroups but not in other renal surgery and ropivacaine subgroups. Despite subgroup analysis, the sources of heterogeneity remain unclear. A recent study indicated that among patients undergoing PCNL, women need more postoperative analgesia than men (37), which might explain the heterogeneity among included studies. Moreover, the implementation methods of PVB of included RCTs were different, as well as the use of the concentration of anesthetics, which might contribute to the high heterogeneity. In addition, this meta-analysis also showed that PVB prolonged the time to first postoperative analgesia requirement and reduced the incidence of postoperative additional analgesia in renal surgery. Subgroup analysis revealed that the first postoperative analgesia time of PVB using bupivacaine was significantly prolonged, which was similar to the results of Saroa et al. (34), indicating that the use of bupivacaine for PVB was more beneficial for pain management of patients undergoing renal surgery. And the reduction in the incidence of postoperative additional analgesia was more significant in PCNL. Based on the current pooled data analysis, we found that the use of PVB for other renal surgery may be less assuring compared to PCNL. None of the RCTs included in this study reported significant complications, and only a few cases of nausea, vomiting, and itching were reported. Furthermore, this study also showed that PVB performed well in the incidence of postoperative nausea, vomiting, and itching. Although the use of PVB for postoperative analgesia in renal surgery has obvious advantages, however, the lack of professional equipment, high technical difficulty, and potential risks might limit the promotion of this technology.

Although this study was conducted in strict accordance with the Cochrane Manual standards, several limitations remain. First, the heterogeneity of primary outcome measures was high. The diversity of renal surgery types, the difference in blocking methods, the type and concentration of anesthetics, and the population composition of each study may be the sources of heterogeneity. Second, the asymmetry of the funnel plot indicated the possibility of publication bias, therefore, the results should be interpreted with caution. At last, the number of included studies and sample size were limited, which needs to be further confirmed by large-sample, multi-center, high-quality, and well-designed clinical studies.

## Conclusion

In conclusion, this study indicates that PVB may provide effective postoperative analgesia in patients undergoing renal surgery, especially in PCNL. Moreover, PVB is a safe analgesic method without severe complications. Limited by the quality of included studies, the aforementioned results still need to be verified by more high-quality RCTs.

## Data Availability

The original contributions presented in the study are included in the article/Supplementary Material, further inquiries can be directed to the corresponding author/s.

## References

[1] Kehlet H. Postoperative pain, analgesia, and recovery-bedfellows that cannot be ignored. Pain. (2018) 159(Suppl 1):S11–S6. 10.1097/j.pain.0000000000001243 30113942

[2] Chou R Gordon DB de Leon-Casasola OA Rosenberg JM Bickler S Brennan T Management of postoperative pain: a clinical practice guideline from the American Pain Society, the American Society of regional anesthesia and pain medicine, and the American Society of anesthesiologists’ committee on regional anesthesia, executive committee, and administrative council. J Pain. (2016) 17:131–57. 10.1016/j.jpain.2015.12.008 26827847

[3] Lovich-Sapola J Smith CE Brandt CP. Postoperative pain control. Surg Clin North Am. (2015) 95:301–18. 10.1016/j.suc.2014.10.002 25814108

[4] Koo CH Ryu JH. Anesthetic considerations for urologic surgeries. Korean J Anesthesiol. (2020) 73:92–102. 10.4097/kja.19437 31842248 PMC7113163

[5] Zimmer A Greul F Meißner W. Pain management in urology. Urologe A. (2013) 52:585–95. 10.1007/s00120-013-3164-y 23529794

[6] Borys M Szajowska P Jednakiewicz M Wita G Czarnik T Mieszkowski M Quadratus lumborum block reduces postoperative opioid consumption and decreases persistent postoperative pain severity in patients undergoing both open and laparoscopic nephrectomies-a randomized controlled trial. J Clin Med. (2021) 10:3590. 10.3390/jcm10163590 34441884 PMC8396843

[7] Bruintjes MHD van Helden EV de Vries M Wirken L Evers AWM van Middendorp H Chronic pain following laparoscopic living-donor nephrectomy: prevalence and impact on quality of life. Am J Transplant. (2019) 19:2825–32. 10.1111/ajt.15350 30868731 PMC6790588

[8] D'Ercole F Arora H Kumar PA. Paravertebral block for thoracic surgery. J Cardiothorac Vasc Anesth. (2018) 32:915–27. 10.1053/j.jvca.2017.10.003 29169795

[9] Albi-Feldzer A Dureau S Ghimouz A Raft J Soubirou JL Gayraud G Preoperative paravertebral block and chronic pain after breast cancer surgery: a double-blind randomized trial. Anesthesiology. (2021) 135:1091–103. 10.1097/ALN.0000000000003989 34618889

[10] Scarfe AJ Schuhmann-Hingel S Duncan JK Ma N Atukorale YN Cameron AL. Continuous paravertebral block for post-cardiothoracic surgery analgesia: a systematic review and meta-analysis. Eur J Cardiothorac Surg. (2016) 50:1010–8. 10.1093/ejcts/ezw168 27242357

[11] Fusco P Cofini V Petrucci E Scimia P Paladini G Behr AU Unilateral paravertebral block compared with subarachnoid anesthesia for the management of postoperative pain syndrome after inguinal herniorrhaphy: a randomized controlled clinical trial. Pain. (2016) 157:1105–13. 10.1097/j.pain.0000000000000487 26761379

[12] You X Liu W. Evaluation of analgesia effect after ultrasound-guided laparoscopic renal surgery. Comput Math Methods Med. (2021) 2021:6194806. 10.1155/2021/6194806 34976111 PMC8719997

[13] Moher D Liberati A Tetzlaff J Altman DG, PRISMA Group. Preferred reporting items for systematic reviews and meta-analyses: the PRISMA statement. PLoS Med. (2009) 6:e1000097. 10.1371/journal.pmed.1000097 19621072 PMC2707599

[14] Higgins JP Altman DG Gøtzsche PC Jüni P Moher D Oxman AD The Cochrane Collaboration's tool for assessing risk of bias in randomised trials. BMJ. (2011) 343:d5928. 10.1136/bmj.d5928 22008217 PMC3196245

[15] Ak K Gursoy S Duger C Isbir AC Kaygusuz K Ozdemir Kol I Thoracic paravertebral block for postoperative pain management in percutaneous nephrolithotomy patients: a randomized controlled clinical trial. Med Princ Pract. (2013) 22:229–33. 10.1159/000345381 23257888 PMC5586743

[16] Akıncı G Hatipoğlu Z Güleç E Özcengiz D. Effects of ultrasound-guided thoracic paravertebral block on postoperative pain in children undergoing percutaneous nephrolithotomy. Turk J Anaesthesiol Reanim. (2019) 47:295–300. 10.5152/TJAR.2019.81205 31380510 PMC6645846

[17] Baik JS Oh AY Cho CW Shin HJ Han SH Ryu JH. Thoracic paravertebral block for nephrectomy: a randomized, controlled, observer-blinded study. Pain Med. (2014) 15:850–6. 10.1111/pme.12320 24341324

[18] Baldea KG Patel PM Delos Santos G Ellimoottil C Farooq A Mueller ER Paravertebral block for percutaneous nephrolithotomy: a prospective, randomized, double-blind placebo-controlled study. World J Urol. (2020) 38:2963–9. 10.1007/s00345-020-03093-3 31982963

[19] Borle AP Chhabra A Subramaniam R Rewari V Sinha R Ramachandran R Analgesic efficacy of paravertebral bupivacaine during percutaneous nephrolithotomy: an observer blinded, randomized controlled trial. J Endourol. (2014) 28:1085–90. 10.1089/end.2014.0179 24828850

[20] Copik M Bialka S Daszkiewicz A Misiolek H. Thoracic paravertebral block for postoperative pain management after renal surgery: a randomised controlled trial. Eur J Anaesthesiol. (2017) 34:596–601. 10.1097/EJA.0000000000000673 28731925

[21] Hatipoglu Z Gulec E Turktan M Izol V Arıdogan A Gunes Y Comparative study of ultrasound-guided paravertebral block versus intravenous tramadol for postoperative pain control in percutaneous nephrolithotomy. BMC Anesthesiol. (2018) 18:24. 10.1186/s12871-018-0479-7 29454333 PMC5816552

[22] Kamble TS Deshpande CM. Evaluation of the efficacy of bupivacaine (0.5%) alone or with clonidine (1 *μ*g/kg) versus control in a single level paravertebral blockin patients undergoing PCNL procedure. J Clin Diagn Res. (2016) 10:UC13–UC7. 10.7860/JCDR/2016/20890.9033 PMC5296552 28208979

[23] Li C Song C Wang W Song C Kong X. Thoracic paravertebral block versus epidural anesthesia combined with moderate sedation for percutaneous nephrolithotomy. Med Princ Pract. (2016) 25:417–22. 10.1159/000447401 27265121 PMC5588437

[24] Maheshwari PN Wagaskar VG Apathak A Garcha PS Mhatre T. A prospective, randomised, double-blind comparative study for efficacy of paravertebral block byropivacaine in postoperative analgesia after percutaneous nephrolithotomy. J Clin Diagnostic Res. (2017) 11:PC20–PC2. 10.7860/JCDR/2017/24378.11017

[25] Moawad HE Mousa SA El-Hefnawy AS. Single-dose paravertebral blockade versus epidural blockade for pain relief after open renal surgery: a prospective randomized study. Saudi J Anaesth. (2013) 7:61–7. 10.4103/1658-354X.109814 23717235 PMC3657929

[26] Narasimhan P Kashyap L Mohan VK Arora MK Shende D Srinivas M Comparison of caudal epidural block with paravertebral block for renal surgeries in pediatric patients: a prospective randomised, blinded clinical trial. J Clin Anesth. (2019) 52:105–10. 10.1016/j.jclinane.2018.09.007 30243061

[27] Yaman F Tuglu D. Analgesic efficacy of ultrasound guided paravertebral block in percutaneous nephrolithotomy patients: a randomized controlled clinical study. BMC Anesthesiol. (2020) 20:250. 10.1186/s12871-020-01169-6 32993528 PMC7523349

[28] Yayik AM Ahiskalioglu A Demirdogen SO Ahiskalioglu EO Alici HA Kursad H. Ultrasound-guided low thoracic paravertebral block versus peritubal infiltration for percutaneous nephrolithotomy: a prospective randomized study. Urolithiasis. (2020) 48:235–44. 10.1007/s00240-018-01106-w 30564847

[29] Yenidünya O Bircan HY Altun D Caymaz I Demirag A Turkoz A. Anesthesia management with ultrasound-guided thoracic paravertebral block for donor nephrectomy: a prospective randomized study. J Clin Anesth. (2017) 37:1–6. 10.1016/j.jclinane.2016.10.038 28235492

[30] Zhang W Wan Z Zhou R Xu K Xiao P Liang Z Application of continuous thoracic paravertebral nerve block guided by nerve stimulator on postoperative pain relief for nephrectomy. Zhonghua Yi Xue Za Zhi. (2014) 94:1812–4. 10.3760/cma.j.issn.0376-2491.2014.23.015 25154849

[31] Ardon AE Lee J Franco CD Riutort KT Greengrass RA. Paravertebral block: anatomy and relevant safety issues. Korean J Anesthesiol. (2020) 73:394–400. 10.4097/kja.20065 32172551 PMC7533185

[32] Lönnqvist PA Olsson GL. Paravertebral vs epidural block in children. Effects on postoperative morphine requirement after renal surgery. Acta Anaesthesiol Scand. (1994) 38:346–9. 10.1111/j.1399-6576.1994.tb03905.x 8067221

[33] Deng J Wei K Li M Wang X Tang Q. Paravertebral block reduces pain in elderly patients with percutaneous nephrolithotomy: a randomized controlled study protocol. Medicine (Baltimore). (2020) 99:e23761. 10.1097/MD.0000000000023761 33371139 PMC7748350

[34] Saroa R Palta S Puri S Kaur R Bhalla V Goel A. Comparative evaluation of ropivacaine and levobupivacaine for postoperative analgesia after ultrasound-guided paravertebral block in patients undergoing percutaneous nephrolithotomy. J Anaesthesiol Clin Pharmacol. (2018) 34:347–51. 10.4103/joacp.JOACP_187_17 30386018 PMC6194850

[35] Tomar GS Ganguly S Cherian G. Effect of perineural dexamethasone with bupivacaine in single space paravertebral block for postoperative analgesia in elective nephrectomy cases: a double-blind placebo-controlled trial. Am J Ther. (2017) 24:e713–e7. 10.1097/MJT.0000000000000405 26938764

[36] Tan X Fu D Feng W Zheng X. The analgesic efficacy of paravertebral block for percutaneous nephrolithotomy: a meta-analysis of randomized controlled studies. Medicine (Baltimore). (2019) 98:e17967. 10.1097/MD.0000000000017967 31770205 PMC6890373

[37] Jonnavithula N Garg H Allenki P Aavula K. Influence of gender on postoperative pain in percutaneous nephrolithotomy: a prospective observational study. J Anaesthesiol Clin Pharmacol. (2021) 37:449–452. 10.4103/joacp.JOACP_314_19 34759560 PMC8562457

