# Supplementary material for: The efficacy and safety of paravertebral block for postoperative analgesia in renal surgery: A systematic review and meta-analysis of randomized controlled trials
Source: Front Surg. 2022 Jul 18;9:865362. doi: 10.3389/fsurg.2022.865362 (PMC9339658; doi:10.3389/fsurg.2022.865362)
Supplement: Supplementary file 1 [file Table_1_v1.docx]

**Supplementary Material**

**Search Strategies:**

**Pubmed**

((("paravertebral"[All Fields] OR "paravertebrally"[All Fields]) AND ("block"[All Fields] OR "blocked"[All Fields] OR "blocking"[All Fields] OR "blockings"[All Fields] OR "blocks"[All Fields])) OR "PVB"[All Fields]) AND ("nephrolithotomy, percutaneous"[MeSH Terms] OR ("nephrolithotomy, percutaneous"[MeSH Terms] OR ("nephrolithotomy"[All Fields] AND "percutaneous"[All Fields]) OR "percutaneous nephrolithotomy"[All Fields] OR ("nephrolithotomies"[All Fields] AND "percutaneous"[All Fields]) OR ("nephrolithotomy, percutaneous"[MeSH Terms] OR ("nephrolithotomy"[All Fields] AND "percutaneous"[All Fields]) OR "percutaneous nephrolithotomy"[All Fields] OR ("percutaneous"[All Fields] AND "nephrolithotomies"[All Fields]) OR "percutaneous nephrolithotomies"[All Fields])) OR ("nephrectomy"[MeSH Terms] OR "nephrectomy"[All Fields] OR "nephrectomies"[All Fields] OR ("nephrectomy"[MeSH Terms] OR "nephrectomy"[All Fields] OR "heminephrectomies"[All Fields] OR "heminephrectomy"[All Fields]) OR ("nephrectomy"[MeSH Terms] OR "nephrectomy"[All Fields] OR "heminephrectomies"[All Fields] OR "heminephrectomy"[All Fields])) OR ("pyeloplasties"[All Fields] OR "pyeloplasty"[All Fields] OR (("renal"[All Fields] OR "renals"[All Fields]) AND ("surgery"[MeSH Subheading] OR "surgery"[All Fields] OR "surgical procedures, operative"[MeSH Terms] OR ("surgical"[All Fields] AND "procedures"[All Fields] AND "operative"[All Fields]) OR "operative surgical procedures"[All Fields] OR "general surgery"[MeSH Terms] OR ("general"[All Fields] AND "surgery"[All Fields]) OR "general surgery"[All Fields] OR "surgery s"[All Fields] OR "surgerys"[All Fields] OR "surgeries"[All Fields]))))

**Embase**

#1 'paravertebral block':ab,ti OR 'pvb':ab,ti

#2 'percutaneous nephrolithotomy'/exp

#3 'nephrolithotomies, percutaneous':ab,ti OR 'percutaneous nephrolithotomies':ab,ti

#4 'nephrectomy'/exp

#5 'nephrectomies':ab,ti OR 'heminephrectomy':ab,ti OR 'heminephrectomies':ab,ti OR 'pyeloplasty':ab,ti OR 'renal surgery':ab,ti

#6 #2 OR #3 OR #4 OR #5

#7 #1 AND #6

**Web of science**

#1 TS=(paravertebral block OR PVB)

#2 TS=(percutaneous nephrolithotomy OR nephrolithotomies, Percutaneous OR Percutaneous nephrolithotomies OR nephrectomy OR Nephrectomies OR Heminephrectomy OR heminephrectomized OR pyeloplasty OR renal surgery)

#3 #1 AND #2

**Cochrane**

#1 (paravertebral block):ti,ab,kw OR (PVB):ti,ab,kw

#2 MeSH descriptor: [nephrolithotomy, percutaneous]

#3 MeSH descriptor: [nephrectomy]

#4 (nephrolithotomies, percutaneous):ti,ab,kw OR (percutaneous nephrolithotomies):ti,ab,kw OR (nephrectomies):ti,ab,kw OR (heminephrectomy):ti,ab,kw OR (heminephrectomies):ti,ab,kw

#5 (pyeloplasty):ti,ab,kw OR (renal surgery):ti,ab,kw

#6 #2 OR #3 OR #4 OR #5

#7 #1 AND #6
